# Obtaining and Characterization of a Polydisperse System Used as a Transmembrane Carrier for Isosorbide Derivatives

**DOI:** 10.3389/fchem.2020.00492

**Published:** 2020-06-30

**Authors:** Florin Borcan, Adél Len, Diana A. Bordejevic, Zoltán Dudás, Mirela C. Tomescu, Adina N. Valeanu

**Affiliations:** ^1^The 1st Department (Analytical Chemistry), Faculty of Pharmacy, “Victor Babes” University of Medicine and Pharmacy, Timisoara, Romania; ^2^Neutron Spectroscopy Department, Centre for Energy Research, Hungarian Academy of Sciences, Budapest, Hungary; ^3^Faculty of Engineering and Information Technology, University of Pécs, Pécs, Hungary; ^4^The 5th Department (Internal Medicine I), Faculty of Medicine, “Victor Babes” University of Medicine and Pharmacy, Timisoara, Romania; ^5^“Coriolan Drǎgulescu” Institute of Chemistry, Romanian Academy, Timisoara, Romania; ^6^The 2nd Department, Faculty of Dental Medicine, “Victor Babes” University of Medicine and Pharmacy, Timisoara, Romania

**Keywords:** chitosan, drug delivery, polyurethane, skin irritation, microstructural characterization

## Abstract

Due to their effect of vasodilatation, isosorbide nitrates represent one of the most important and most used solutions for angina pectoris. Unfortunately, these compounds have multiple dose-related adverse drug reactions such as headache, weakness, mild dizziness, and occasionally heart rate changes, nausea, vomiting, and sweating. The main aims of this research were to obtain and to evaluate new polyurethane (PU) structures that can be used as a proper transmembrane carrier with an improved release kinetic. Chitosan-based PU structures were obtained by a polyaddition process between hexamethylene diisocyanate and a mixture of chitosan, butanediol, and polyethylene glycol in the presence of caffeine as a synthesis catalyst. The obtained samples (with and without isosorbide nitrates) were characterized regarding the encapsulation and release rate (UV-Vis spectra), chemical composition (FTIR), thermal stability (thermal analysis), morphology changes (SEM and SANS), and *in vivo* irritation tests. These methods revealed no significant differences between the two sample structures. Multipopulational structures with sizes between 73 and 310 nm, with an increased tendency to form clusters and a high resistance to heat (up to 280°C), were obtained. This study presents an alternative administration of isosorbide derivatives based on a PU carrier with a high biocompatibility and a prolonged release.

## Introduction

Nitrate medications (nitroglycerin, sodium nitroprusside, isosorbide derivatives, etc.) are pharmaceutical agents with vasodilator effect (Polakowska et al., 2016). Thus, medication with isosorbide nitrates as active compounds is often used to prevent angina attacks and in the prophylactic treatments of angina pectoris (Yao et al., 2015). They can be also used in combination with cardiotonic glycosides, diuretics, and ACE inhibitors as adjuvant treatment of congestive heart failure (Kocyigit et al., 2017). Isosorbide nitrates include isosorbide dinitrate (IS-DN), 2-mononitrate (IS-2-MN), and 5-mononitrate (IS-5-MN), the last two being active metabolites of IS-DN and they exert qualitatively similar effects. A comparative study on these compounds reveals that IS-2-MN induces a lower increase of cyclic guanosine monophosphate (cGMP) and less tolerance induction, and its vasodilator effect is probably due to other mechanisms than the stimulation of guanylate cyclase (Raddino et al., 2010). U. Thadani and T. Rodgers reported a series of problems due to the administration of isosorbide nitrates: 82% of patients accused headaches and ~10% could not tolerate them due to dizziness and/or disabling headaches; severe hypotension cases accompanied by syncope were recorded; the combination of nitrates and phosphodiesterase-5 inhibitors used together for the treatment of erectile dysfunction induces a major decrease of blood tension and might lead to death (Thadani and Rodgers, 2006).

The particles used as carriers of isosorbide derivatives have a very long history: Hirayama et al. (1988) described three decades ago the obtaining and the physicochemical properties of a carrier, based on heptakis(2,6-di-O-ethyl)-beta-cyclodextrin whose dissolution and IS-DN release rates were significantly decreased after the complexation with the cyclodextrin. Microcapsules based on hydroxypropyl cellulose with three different particle populations were developed by Yang et al. (2006) in order to obtain an improved kinetic model. A possible synergistic effect of IS-5-MN liposomalization and addition of glycerol in a transdermal carrier was studied *in vitro* by Barichello et al. (2017); they have applied both liposomal and aqueous solution with and without glycerol and they have found that glycerol facilitates the skin permeation while the liposomalization process leads to the drug accumulation. Most recently, the research team of Steinbach et al. (2017) has developed microemulsions with ceramide nanoparticles containing isosorbide and they have assessed the retarder action *ex vivo* in a Franz diffusion cell. Their results proved that ceramide particles present a better penetration in the upper layers of the skin without any irritating effect.

The usual routes for drug administration (oral, inhalable, intramuscular/intravenous, topical, and rectal) are selected depending on the drug formula, its toxicity and metabolic pathway, and the part of the body that is treated (Turner et al., 2011). Unfortunately, these classical routes are not always effective to assure the optimal dose of every drug to its specific receptor; there are several important drugs (e.g., hormones) with dosage inside the body whose gradual release is often a problem. The development of drug delivery systems, based on recent nano-sciences developments and on improved knowledge about the body membranes, represents a revolutionary advantage for many medications. Nowadays, organic and inorganic carriers are synthesized to solve two main problems: to avoid the adverse events (the side effects) and to modify the physicochemical properties of drugs in order to improve their transmembrane transfer (Solano-Umana et al., 2015).

The advantage of a polydisperse system used as a drug carrier consists in the gaining of a balance between the endocytosis-dependent cellular uptake and the amount of the encapsulated therapeutic compound; different pathways such as clathrin-caveolin-independent, caveolin-dependent, and clathrin-mediated are specific to nanoparticles, while the particles over 100 nm use macro-pinocytosis and phagocytosis to transfer their load (Danaei et al., 2018). On the other hand, the degradation of particles and their release rate depend on the type of carrier, the loading capacity, and the length of polymer chains. A polydisperse system containing particles with different sizes behaves rather like a slow-release delivery system. The interest for the prolonged released formulations containing isosorbide nitrates started more than four decades ago; R. Bonn has obtained capsules with isosorbide mononitrate that ensures a long-lasting effect (Bonn, 1988). Another long-acting formulation based on a controlled membrane principle was developed after 10 years (Prakash and Markham, 1999) and a comprehensive review describing the need of a prolonged release for this class of drugs was published in the same year (Gunasekara and Noble, 1999).

Polyurethanes (PUs) appeared in the laboratory of Prof. Otto Bayer at IG. Farbenindustrie Leverkusen (Germany) almost one century ago (Gama et al., 2018). There are many types of PU-based materials that look and behave very differently from each other. The main areas of their application are as follows: buildings (thermal insulation, sandwich panels, rigid columns, and other elements of architectural design), refrigeration (thermal insulation of refrigerators and freezers), automotive industry (car dashboard and steering wheel, door panels), furniture (cabinets, elastic mattresses, and pillows), textiles (camping products, adhesives for footwear), etc. Early use of PU in medicine was reported by Boretos and Pierce; they observed its vascular acceptability in experimental heart-assist pump chambers and arterial cannulae (Hasirci and Aksoy, 2007). In the last four decades, the use of PU covers many other medical fields such as cardiovascular devices (catheters, vascular prostheses, pacemakers), reconstructive surgery materials (wound dressings, breast implants, maxillofacial prostheses), and obstetrics and gynecology (condoms, contraceptive sponges; Vermette and Griesser, 2001). Basu et al. (2016) present a series of drug carriers based on PUs: nanoparticle systems, stimulus-responsive systems, shape-memory systems, etc.

The development of a poly(tetramethylene glycol)/isosorbide-based PU elastomer has been reported by Kim et al. (2014). It was found that this highly elastic and biocompatible PU presents increased mechanical properties and it can be used for soft tissue augmentation and regeneration. A similar study describing a PU with self-healing properties (Kim et al., 2019) has revealed that the replacement of traditional chain extenders (such as 1,4-butanediol or 1,6-hexanediol) with isosorbide or isomannide leads to materials with improved mechanical properties. Our research team already synthesized PU nano- and micro-structures with low release rates used as transmembrane delivery systems for different natural extracts (Munteanu et al., 2017; Borcan et al., 2018a,b,c, 2019). The aim of this study was to obtain and to characterize a chitosan-based PU that can assure a constant release of isosorbide derivatives for a prolonged time.

## Materials and Methods

### Reagents

Chitosan-based PU structures were obtained using the following reagents: hexamethylene diisocyanate (HDI), polyethylene glycol (PEG 200), and caffeine were obtained from Merck (Darmstadt, Germany); acetic acid 96%, acetone, and HCl 1N were obtained from Chimopar SA (Bucharest, Romania); 1,4-butanediol (BD) was purchased from Carl Roth GmbH (Karlsruhe, Germany); and chitosan was from Oxford Lab. Chem. (Vasai-Virar, Maharashtra, India). These reagents were of commercial reagent grade; they were kept under the conditions indicated by the manufacturer and they were used without further purifications.

Inorganic salts such as NaCl, KCl, NaHCO_3_, Na_2_HPO_4_, K_2_HPO_4_, KH_2_PO_4_, and MgCl_2_ were purchased from Chimopar SA (Bucharest, Romania), and they were used to prepare a simulated body fluid; they were of analytical grade and they were previously heated to remove the crystallization water.

### Synthesis of Chitosan-Based PU Structures

The first step was the solubilization of chitosan: 100.0 mg chitosan, 2.5 ml acetic acid, and 5 ml HCl were added to 50 ml of double deionized distilled water, and they were mixed on a Velp hot plate stirrer with 300 rpm, at 45°C for 10 min. In the second step, the aqueous phase was prepared by using the entire previously obtained chitosan solution and 2.0 ml of BD, 3.0 ml of PEG 200, and 40.0 mg of caffeine as catalyst of PU synthesis. This mixture was further sonicated with a powerful lab homogenizer (Hielscher UP200S) for another 15 min, and after that, it was split into two different 100-ml polymerization flasks in equal volumes. Ten milliliters of aqueous solution containing 3 mmol IS-2-MN, 1 mmol IS-5-MN, and 5 mmol IS-DN was added in one of the flasks, and it was labeled as PU_1 (sample containing isosorbide nitrates), while the other flask was labeled PU_0 (reference sample, empty chitosan-based PU without nitrates).

In the third step of the synthesis, the organic phase was prepared in a 100-ml Berzelius beaker: 14.0 ml of HDI was dissolved in 50 ml of acetone, and the mixture was homogenized for 10 min and was poured in the two polymerization flasks in equal volumes.

The content of these flasks was stirred with 300 rpm, at room temperature for 90 min in order to complete all chemical reactions of macromolecular chains.

In the last step, the synthesis products were washed three times with a mixture of acetone and water (1:1, v/v) and they were slowly dried by being kept as thin layers at room temperature till no mass change was observed.

**Figure 1:**
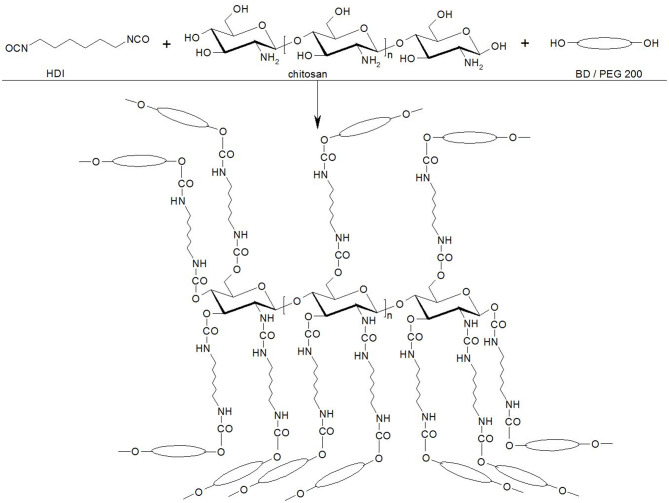
The chemical reactions between the main raw materials.

Figure 1 presents the starting chemical reactions that happen around the chitosan molecules and lead to a three-dimensional growth of chains.

### Drug Encapsulation Efficiency

**Figure 2:**
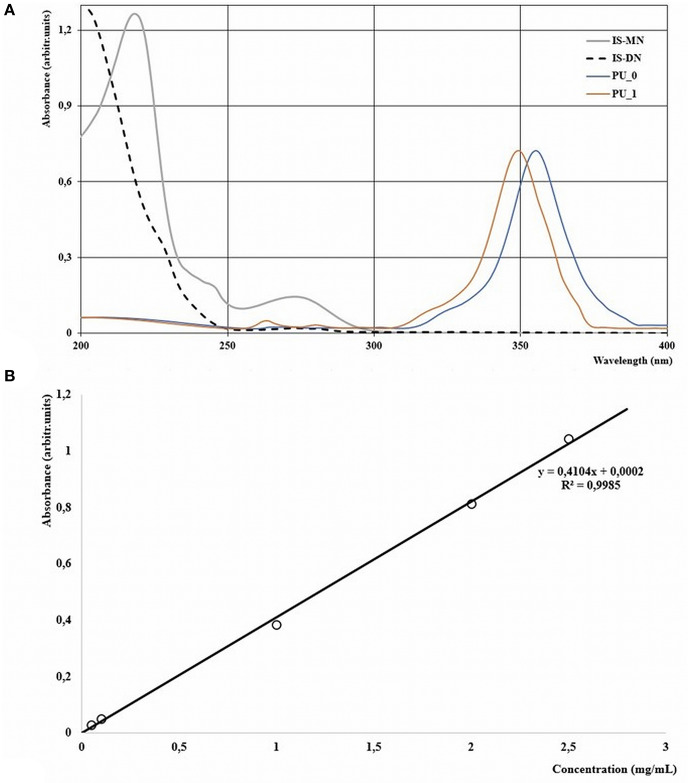
**(A)** UV-Vis spectra of samples and **(B)** calibration curve of isosorbide mononitrate.

The percentage of drug that was successfully entrapped inside a carrier can be calculated by reporting the free/untrapped drug to the total amount of drug added (Bouchemal et al., 2004). Figure 2 presents the UV spectra of isosorbide nitrates and of PU structures obtained on a UVi Line 9,400 Spectrophotometer (SI Analytics, Germany). It can be observed that IS-DN has no maximum peak, while the two isosorbide mononitrate isomers' (IS-MN) spectra overlap perfectly. A calibration curve, describing the dependence between the absorbance at 220 nm and the concentration of IS-2-MN standard solutions, was drawn in accordance with Tsai et al. (1994).

### Drug Release Kinetics

The cumulative percentage of isosorbide derivatives that were released at a specific moment was determined by maintaining the chitosan-based PU structures with derivatives (sample PU_1) in a degradation medium (a simulated body fluid, SBF according to T. Kokubo recipe; Quan et al., 2016) for 3 weeks; the procedure is described in detail in one of our previous papers (Borcan et al., 2018c).

### Thermal Analysis

The resistance of samples to heating and the study of thermal degradation processes were done using a DSC1 (Mettler-Toledo, Switzerland); 4 ± 0.3-mg samples were placed in aluminum crucibles with pierced lids, and they were heated in an inert atmosphere between 30 and 300°C with 5°C/min.

### Zetasizer Measurements

The size and the surface charge of the chitosan-based PU structures with and without isosorbide derivatives were assessed using a Cordouan Zetasizer (Cordouan Technol., France). The following input parameters of the size module were set: evaluation temperature (25°C), time intervals (12 μs), number of channels (225), laser power (80 ± 5%), acquisition mode (statistical mode with noise limit; minimum 6 acquisitions/sample), and analysis mode (Cumulants). In the case of Zeta potential module, the parameters were quartz cuvette, temperature (25°C), laser power (75 ± 5%), applied field (automatic), medium resolution, 3 measures/sequence, and Henry function (Smoluchowski).

### Scanning Electron Microscopy (SEM)

The shape and surface morphology of the PU nano-/micro-particles were determined from micrographs recorded with a TESCAN 3 VEGA scanning electron microscope secondary electron detector. The high-voltage value was set at 20 kV. The samples were tested in their natural form under vacuum.

### Fourier Transform Infrared Spectroscopy (FTIR)

Differences on the chemical composition of the two samples' functional groups were analyzed by FTIR spectroscopy. Hereby, FTIR spectra were collected, using KBr pellets, through the use of a Cary 630 FTIR spectrophotometer (Agilent, USA), in the wavenumber ranging 400–4,000 cm^−1^.

### Small-Angle Neutron Scattering (SANS)

SANS measurements were performed on the Yellow Submarine (YS) instrument at the Budapest Neutron Centre in Hungary. Neutrons with wavelengths of 4.2 and 10.2 Å and sample-to-detector distances of 1.15 and 5.25 m were chosen. This range of wave vectors corresponds to structural sizes ranging from 1 to 100 nm. The samples were deposited in quartz cuvettes with 2-mm thickness and they were placed for 30 to 60 min in an 8-mm-diameter neutron beam.

### *In vivo* Evaluations

Seven human volunteers (3 men and 4 women, between 25 and 37 years old) were used to evaluate the skin irritation caused by the products. The two synthesized samples (PU_0 and PU_1) and a mixture of isosorbide derivatives as reference were applied as solutions (1:20 w/v) on three areas of the left anterior forearm every third day (0.5 ml/application) and the determinations of the skin parameters were performed 25 min later on every treated area. The measurements were carried out with a MultiProbe Adapter from Courage&Khazaka Electronics (Köln, Germany), equipped with a Tewameter® TM300 probe and a Mexameter® MX18 probe, by the same operator, at the same moment of the day for 15 days. The values are expressed as differences between after and before any application due to the differences in skin types of the analyzed volunteers.

### Statistics and Ethical Approval

The statistical analyses were performed using IBM SPSS v.23; the results were expressed as mean ± standard error. One-Way ANOVA and Bonferonni–Dunn tests were used to determine the statistical difference between experimental and blank values groups; ^**^ and ^***^ indicate *p* <0.01 and *p* <0.001 on **Figures 11**, **12**.

The research was done according to the principles of the Declaration of Helsinki. The authors declare that all procedures respect the specific regulations and standards: the study was first evaluated and approved by the Ethical Committee of “Victor Babes” University of Medicine and Pharmacy Timisoara, Romania. Every volunteer read and signed an informed consent and a signed informed consent for publication of the research results was also obtained.

## Results

Three-dimensional PU structures are often found among those materials that are preferably used in medical applications due to their easy and cheap production as well as to their unique combination of biocompatibility, sterilizability, durability, good mechanical properties, abrasion, and chemical resistance (Kikuchi et al., 2017).

### Drug Encapsulation Efficiency

The difference between the most intense absorption positions of the empty PU structures (360 nm) and isosorbide mononitrates (220 nm) represents an important advantage in the evaluation of the concentration of the active substances in the studied solutions. Encapsulation efficiency equal to 72.3% was found by reporting the quantity of free mononitrates inside the washing mixture from the last synthesis step to the total amount of isosorbide mononitrates added to synthesis.

### Drug Release Kinetics

**Figure 3:**
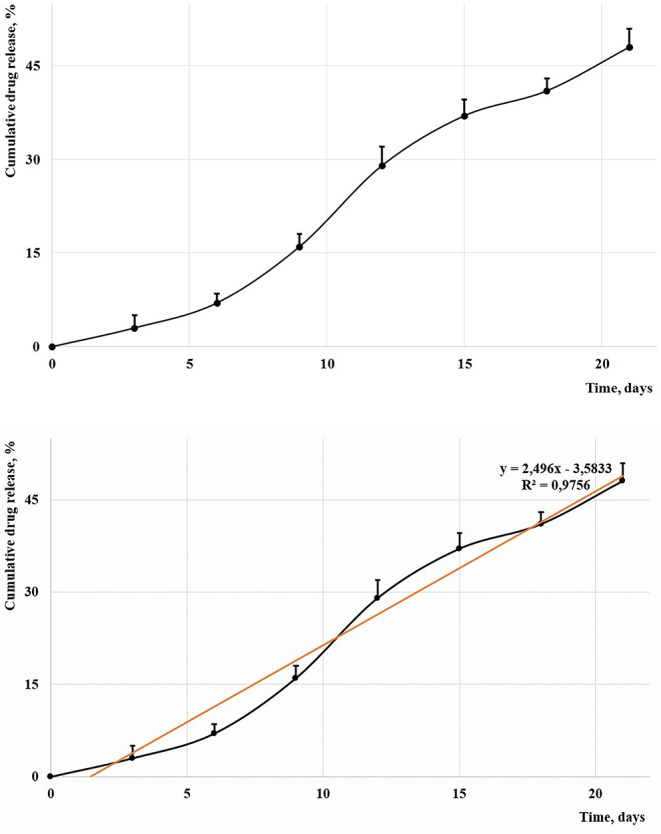
The cumulative release of isosorbide derivatives.

Figure 3 presents the evolution of the cumulative percentage of isosorbide mononitrates that were released inside a simulated body fluid; 5 aliquots of each 0.4 ml were replaced every third day with fresh medium, and their maximum absorption was found at 220 nm; mean concentrations and standard deviation were used to explain the release profile.

### Thermal Analysis

**Figure 4:**
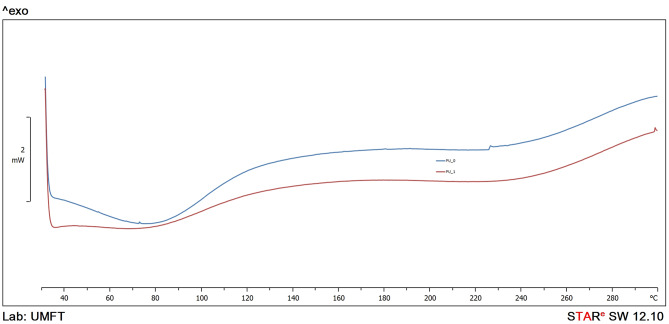
DSC thermograms of chitosan-based PU structures.

DSC curves (Figure 4) indicate a mild dehydration process during the samples' heating between 50 and 100°C (a large endothermic peak) and a good thermal stability up to 260–280°C. No important difference was observed between the samples and no melting point of isosorbide derivatives was found, which indicates the absence of the isosorbide derivatives in their free form.

### Zetasizer Measurements

**Figure 5:**
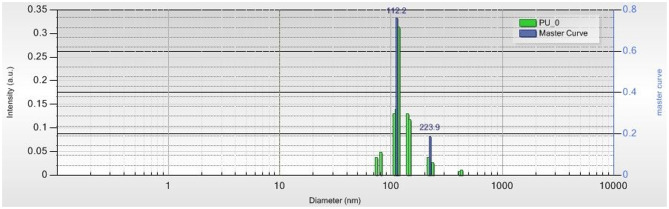
The size distribution of structures from sample PU_0.

**Figure 6:**
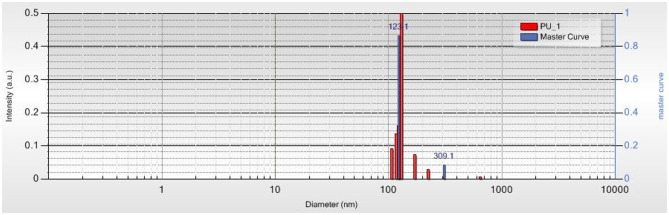
The size distribution of structures from sample PU_1.

Figures 5, 6 present the distribution of PU structure size. The complexity and multipopulation character of the samples are confirmed by their polydispersity index (PDI). Master curve, a very precious indicator for stable polydisperse samples, obtained using Pade-Laplace analysis, is defined by the averaged autocorrelation function over the whole run. PDI for sample PU_0 was found as 0.7 with five different structure populations between 73 and 408 nm. Predominant populations were 112.2 nm (76.2%) and 223.9 nm (18.6%). PDI for sample PU_1 was found as 0.6 with four main populations between 104 and 310 nm. Predominant populations were 123.1 nm (86.4%) and 309.1 nm (8.3%). Zeta potential values determined as the mobility of structures in an electric field were 10.71 mV (sample PU_0) and 9.55 mV (sample PU_1), values that are specific to colloidal systems with a high tendency to form agglomerations of structures.

### Scanning Electron Microscopy (SEM)

**Figure 7:**
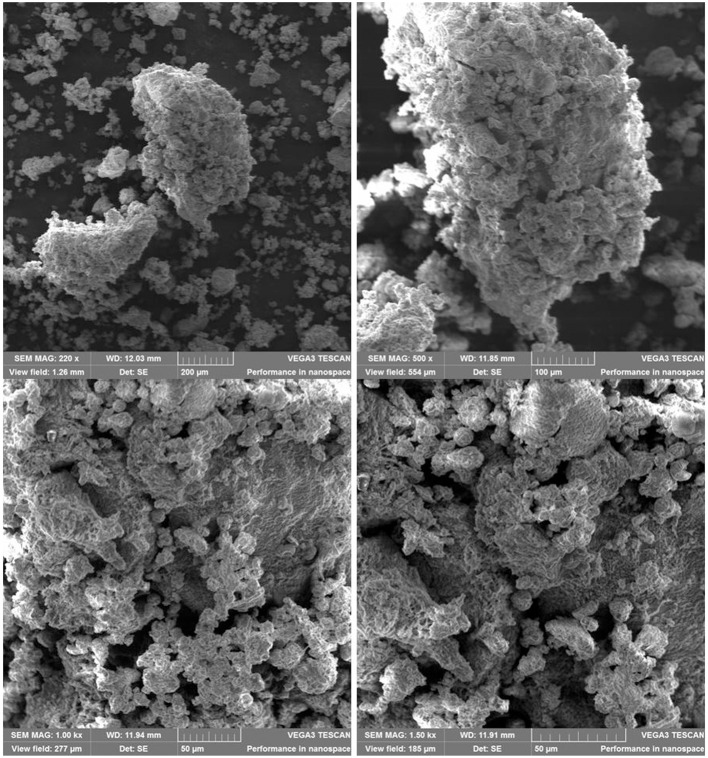
Aspect of structures from sample PU_0 at different magnifications.

**Figure 8:**
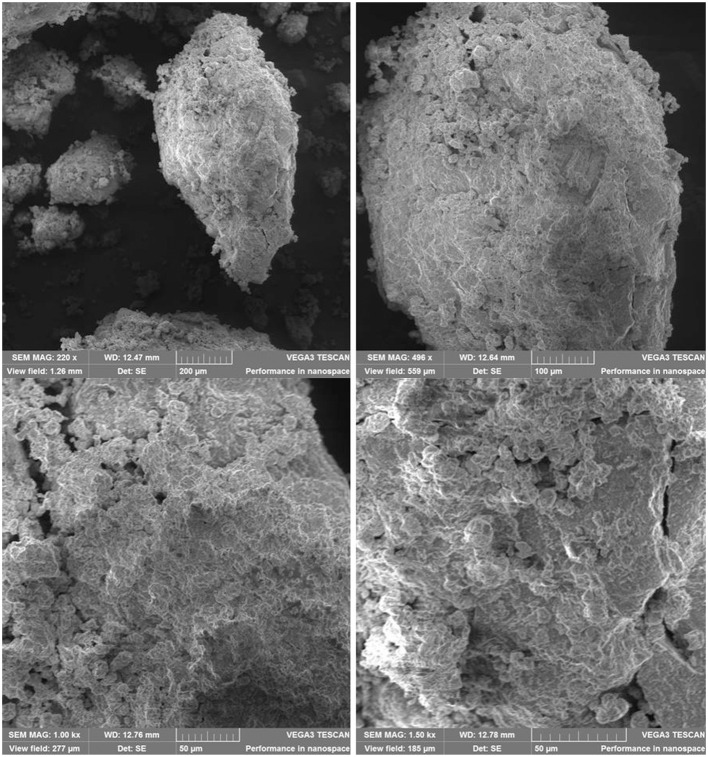
Aspect of structures from sample PU_1 at different magnifications.

The aspect of samples studied by SEM at different magnifications (220 × , 500 × , 1,000 × , and 1,500 × ) confirms the desired heterogeneity of chitosan-based PU structures (Figures 7, 8) without an explicit difference between the PU_0 and PU_1 samples.

### Fourier Transform Infrared Spectroscopy

**Figure 9:**
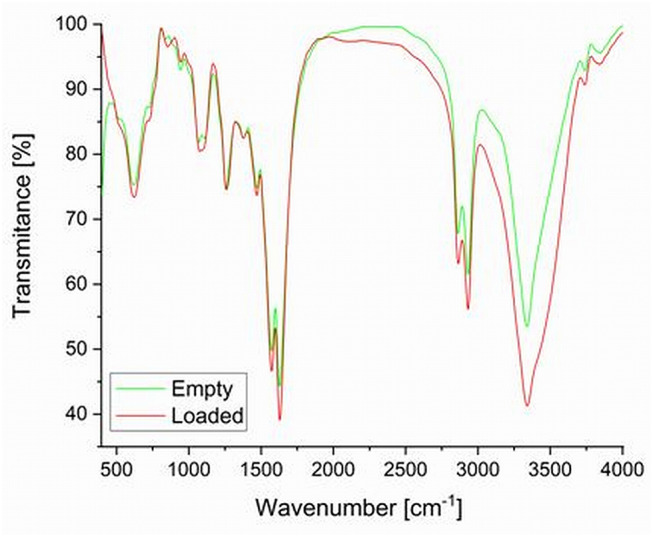
Comparative FT-IR spectra of empty and drug-loaded PU capsules.

FTIR is one of the most important analysis methods in this domain. The diversity of PU functional groups justifies its use. PUs contain multiple urethane groups (-NH-CO-O-), but they are in balance with others: free hydroxyl (-OH) and amine rests (-NH_2_, -NH-) or functional groups specific to secondary products as allophanate (-NH-CO-NR-CO-O-), biuret (-NH-CO-NR-CO-NH-), and urea (-NH-CO-NH-). Figure 9 presents the overlapped IR spectra obtained for the two samples.

### SANS Analysis

**Figure 10:**
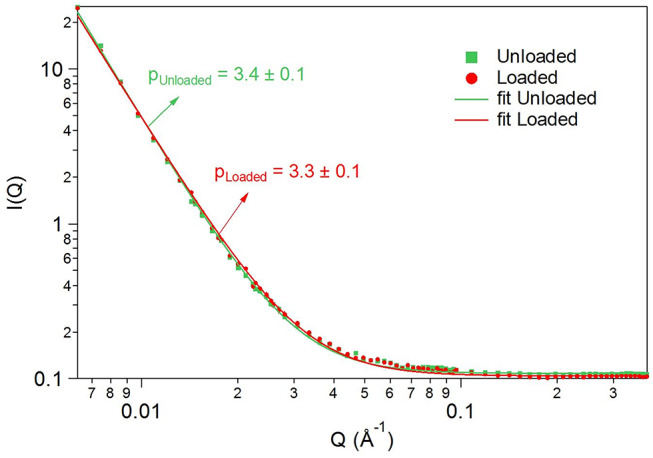
Comparative SANS analysis.

SANS method was used to evaluate the microtextural properties of chitosan-based PU structures. Figure 10 presents comparative information about the synthesized samples. The calibrated SANS data curves were described with a power-law type scattering, according to the Porod approximation (see Equation 1), used for large scattering objects (where *QR* >> 1, *R* being a characteristic, average size of the scattering particles).

(1) $$\begin{array}{l} I\left(Q\right)=AQ^{-p}+B \end{array}$$

*A* is a constant containing instrumental attributes and sample physical characteristics, and *B* is the incoherent background scattering. In this *Q* vector range, SANS did not give information about the size of the scattering particles/pores/inclusions; however, it characterized their surface. The value of the *p* power was characteristic to rough, fractal-like surfaces and interfaces.

### *In vivo* Evaluations

**Figure 11:**
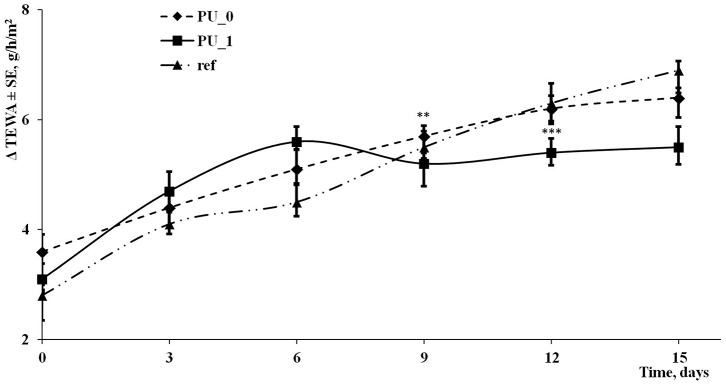
Evolution of TEWA during the experiment.

**Figure 12:**
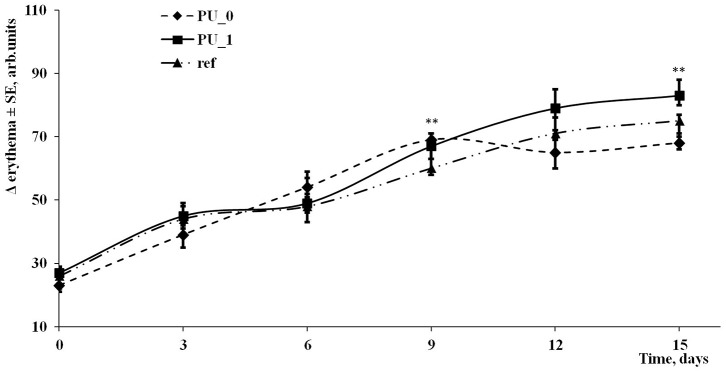
Evolution of erythema during the experiment.

Animal and human skin models are often used to evaluate the irritation effects of new compounds. Modern and non-invasive techniques were developed to assess the skin parameters such as skin pH, transepidermal water loss (TEWA), melanin and erythema, the level of stratum corneum hydration, sebum, etc. Figures 11, 12 present the changes of transepidermal water loss and erythema levels as main indicators of any irritation effect.

## Discussion

In the present synthesis of the PU structures, HDI was used as a diisocyanate instead of aromatic compounds often used in industrial applications for their better mechanical properties. Studies on PU materials degradation revealed that the main raw materials (polyols and isocyanates) were usually obtained during their decomposition (Bolcu and Borcan, 2005); thus, in the present study, an aliphatic diisocyanate has been chosen in order to avoid the introduction of certain aromatic compounds in the human body. Caffeine was used as synthesis catalyst instead of other tertiary amines [1,4-diazabicyclo(2.2.2)octane], tin, or bismuth organic compounds (dibutyl-tin-dilaurate, bismuth neodecanoate; Guhl, 2008); this natural cardiac and central nervous system stimulant was chosen because of its low toxicity. Chitosan has been chosen as a crosslinking agent based on the information about its natural source, ability to swell, capacity to absorb water, and grafting efficiency.

**Figure 13:**
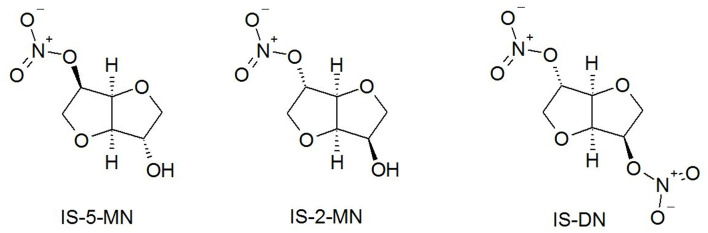
Chemical structures of isosorbide derivatives.

The prolonged release of isosorbide derivatives was obtained by using polyethylene glycol as raw material instead of any other ester polyol that has a faster hydrolysis (Hajnal et al., 2016) and by using a mixture of mono- and di-nitrates (Figure 13): IS-DN does not contain any active hydrogen and it is just physically entrapped inside the polymer matrix, while IS-2-MN and IS-5-MN contain a hydroxyl group and an important part of their amount is covalently bound to the PU chains. Literature describes the extended release as a slower delivery of the entrapped drugs at therapeutic level for 8–12 h while a prolonged release indicates a delivery over a longer and delayed period; Korsmeyer–Peppas model is often used to describe the drug release from polymeric systems (Kakar et al., 2014). Mathematical models have a key role in the interpretation of release profiles; the trend of the present release can be described by the equation *y* = 2.496*x*−3.5833 (*R*^2^ = 0.9756), which is specific to a better release than those described in the case of three nano-fibrous scaffolds based on Nylon 6 or polycaprolactam and used as carrier for Cetirizine (Saadatmand et al., 2019).

Bolcu and Borcan (2005) have reported that PU materials are thermally stable up to 300–320°C depending on the aliphatic/aromatic character of diisocyanates. The insertion of chitosan in the synthesis and the appearance of allophanates in the reaction between PUs' -NH- and an isocyanate, substitute biuret structures in the reaction between -NH_2_ and two isocyanate molecules, amides (carboxylic with isocyanate groups), and urea groups in the reaction between primary amines and one isocyanate group, lead to a decreased thermal stability (the degradation begins at 260–280°C, Figure 4).

The polydispersity, or size heterogeneity of the samples, measured as PDI is a representation of the distribution of different particle populations; PDI is a number between 0.0 for perfectly homogeneous samples and 1.0 for highly heterogeneous samples with multiple particle populations. Carriers with high PDI were extensively employed to enhance the drugs' bioavailability and their release. Danaei et al. (2018) explained a few advantages of drug delivery systems with bigger PDI: the particles of a polydisperse system reach the targeted receptor at different moments and the accumulated effects specific to monodisperse carriers are thus avoided; particles with different sizes have a different capacity to entrap drugs and different degradation and release rates. The Zetasizer measurements confirm the obtaining of multipopulational PU structures that were observed in SEM images (Figures 7, 8), too.

The main functional groups of PU present the following positions of their vibrations in the FTIR spectra: free hydroxyl (3,610–2,870 cm^−1^), -NCO (2,280–2,260 cm^−1^), urethane groups (1,715–1,650 cm^−1^), and allophanate at 1720 cm^−1^ (Bolcu and Borcan, 2005). FTIR spectra of our samples PU_0 and PU_1 (Figure 9) are quite similar with a few little differences. This similarity is due to the good isolation of encapsulated isosorbide derivatives on the one hand and to a good wash of free un-encapsulated raw materials during the last step of the synthesis on the other hand. Specific signals of N-H stretching vibrations can be observed at 3,341–3,339 cm^−1^, stretching vibrations of -CH_2_- and -CH_3_ at 2,932–2,862 cm^−1^, stretching vibrations of C=O from urea and urethane groups at 1,629–1,572 cm^−1^, -CH_2_- deformations at 1,471–1,377 cm^−1^, stretching vibrations of urethane C–O at 1,260-1,256 cm^−1^ and of ether C–O–C between 1,110 and 1,070 cm^−1^, while the other signals correspond to deformation vibration of urethane CO–O bonds. There were no observed specific signals of free -NCO (stretching vibrations at 2,260 cm^−1^) and C = O of isocyanurate rings (1,690 cm^−1^).

The significant absorption bands of isosorbide nitrate FTIR spectra were already described in literature (Silvieri and DeAngelis, 1975): stretching vibrations of aliphatic C–H (2,950–2,850 cm^−1^), stretching vibrations of asymmetric NO_2_ (1,665 and 1,635 cm^−1^), scissoring vibrations of methylene (1,460 cm^−1^), stretching vibrations of symmetric NO_2_ (1,285-1,270 cm^−1^), stretching vibrations of asymmetric C–O–C (~1,100 cm^−1^) and stretching vibrations of O–NO_2_ (865 cm^−1^).

The surface fractal-like character of the studied PU samples nanostructure in the available *Q* scattering vector [*Q* = (4π/λ) sinΘ; λ, neutron wavelength; 2Θ, scattering angle] range (0.006–0.300 Å^−1^) has been shown by SANS. The small-angle scattering curves of both (loaded and unloaded) samples showed power exponents: *p*_loaded_ = 3.3 ± 0.1 and *p*_unloaded_ = 3.4 ± 0.1 (see raw data and fitted models in Figure 10) corresponding to a network-like three-dimensional porous PU skeleton constructed of units with sizes larger than the nanometric scale range, having a rough surface and thus a large surface area. Complementary to the results obtained by SEM, where the texture of the material has been studied on a micrometric level, SANS evidenced surface fractal-like character for both samples at a smaller size scale (size range approximately 20–1,000 Å) in the whole studied volume. This proves that the PU nanostructure was not affected by the drug encapsulation.

The evaluation of skin irritation potential represents a new alternative method to assess the hazard of a new synthesized compound. The skin sensitivity is a very good and fair parameter used in many toxicity assays on new cosmetics, but it can be generally used in the evaluation of any chemicals. The changes of skin parameters (especially the level of transepidermal water loss and erythema) are fast and directly proportional with the irritation potential of the compounds (Kose et al., 2018). The visual observations were replaced by modern, more accurate, credible and non-invasive techniques. In this 2-week experiment, TEWA differences around 6 g/h/m^2^ were observed after the application of multiple samples, and according to Oestmann et al. (1993), these changes were inside a normal range. Erythema measurements indicated an increase by about 70 units after 15 days; increases of erythema levels were seen in every experiment done in our Phyto-science Research Center either in mice or in human skin experiments in the last 8 years due to the skin sensitivity.

Sodium lauryl sulfate (SLS) is often used as control sample in these irritation potential tests. V.G. Gurita (Ciobotaru) and her team have reported increases of TEWA around 11 g/h/m^2^ and of erythema index approximately 90 units using the same instruments in a 25-h experiment on the skin of healthy human volunteers (Gurita (Ciobotaru) et al., 2019). In another study of our team, SLS solution was used as reference in an assessment of the toxicological profile of betulin entrapped in a PU carrier (Borcan et al., 2018c); they presented comparative values of TEWL and erythema between the PU samples and SLS at 6 days, and important increases were found in the case of the well-known irritative agent (around 4 g/h/m^2^ for the transepidermal water loss and more than 175 units for erythema index).

In conclusion, PU structures were developed by using a polyaddition process between chitosan, an aliphatic isocyanate, and an ether polyol as main raw materials. The obtained polydisperse carrier, consisting of structures between 104 and 310 nm, presents good encapsulation efficiency of isosorbide derivatives (around 70%), a very slow release, and a good stability against thermal degradation. The carrier structures possess low values of their surface charge, which indicates a high tendency to form clusters, confirmed by SEM images; their maximum absorption was recorded at 345–360 nm. The skin irritation potential of samples, measured on human volunteers as transepidermal water loss and erythema level, did not show important modifications of these parameters in a 15-day experiment.

## Data Availability Statement

All datasets presented in this study are included in the article/supplementary material.

## Ethics Statement

The studies involving human participants were reviewed and approved by Ethical Committee of Victor Babes University of Medicine and Pharmacy Timisoara, Romania. The patients/participants provided their written informed consent to participate in this study.

## Author Contributions

Conceptualization: FB, ZD, and MT. Methodology and investigation: AL and DB. Software: MT. Resources: FB and AV. Writing—original draft preparation: FB and ZD. Writing—review and editing: FB, AL, and MT. Project administration: FB. All authors contributed to the article and approved the submitted version.

## Conflict of Interest

The authors declare that the research was conducted in the absence of any commercial or financial relationships that could be construed as a potential conflict of interest.
